# Psoric Theory

**Published:** 1883-11

**Authors:** A. McNeil

**Affiliations:** Jeffersonville, Ind.


### PSORIC THEORY.

A. McNeil, M. D., Jeffersonville, Ind.

On the two hundred and ninety-third page of the Homœopathic Physician I translated a part of the lecture of a distinguished French dermatologist, in which he took, substantially, the same ground as Hahnemann's Psoric Theory. I add the views of other distinguished French dermatologists. I first translate Bazin's definition of "Arthrisme:" "A constitutional, not contagious, disease, which is characterized by the frequent production of tophus on the joints and of different eruptions on the skin. They are either dry or moist; the former are circular, isolated, unsymmetrical, multiple, are seated principally on uncovered parts, do not itch, alternate with affections of the joints, disappear in the fourth (last) stage, to be succeeded by visceral complications; to it belongs—*a.* pseudo-exanthems: erythema nodosum, urticaria, pityrasis rubra, herpes zoster, pemphigus 'arthriticus;' *b.* dry arthritides: erythema papulo-tuberculosum, intertrigo, cnidosis, acne rosacea, pityriasis, psoriasis, prurigo, lichen, acne 'arthrica;' *c.* moist arthritides: hydrou, eczema, pompholyx, sycosis, ecthyma, furunculi herpitigueza."

He also defines the herpetic diathesis (herpitides). It is a hereditary, constitutional disease, neither contagious nor communicable by inoculation, with phenomena on the skin and mucous membranes which may be supplanted by diseases of the viscera. When on the skin, it is characterized by obstinacy, long continuance, universality, and tendency to relapse. To it belongs the pseudo-exanthemeta: roseola, urticaria, pityriasis rubra "herpetica," eczema rubrum universale, herpes zona herpetica, pemphigus acutus; dry herpes: cnidosis, epinyctis, pityriasis, psoriasis, prurigo, all lichens qualified by the adjective "herpetic;" moist herpes: eczema, pompholyx, melitagra, ecthyma, furunculi ("herpettiques.")

The following is in the latest and most extensive French work in dermatology, viz.: *Hillairet Traite des Malad de Peaubascic*, page 157: "The treatment of arthritic forms of disease, particularly in the aged, must be carried on with extreme caution. We were the witnesses of the following occurrence: An aged man of eighty suffered from varix complicated with eczema, attended with violent itching. He employed an energetic treatment by washing with solution of corrosive sublimate; he then recovered; but soon afterward he was attacked with inflammation of the lungs and gout. By the employment of mustard-plasters, the eczema was recalled to the skin, the pneumonia disappeared, and the gout soon went also. *Some time after the patient treated himself for the eczema*, pleuritis and endocarditis set in, and apoplexy carried him off in a few days." On page 179, *loc. cit*, the same author reports that he had once seen epilepsy and another time acute tuberculosis appear after the rapid healing of psoriasis.

I have shown the opinions of four of the most eminent authorities on dermatology in France—allopathic, of course—proving that they believed, with Hahnemann, that it is injurious to suppress diseases on the skin by external applications. This is the essence of the Psoric Theory. And yet men calling themselves his followers treat it with derision and go on doing what the progressive men in the old school condemn as dangerous to health, and even life. I hope they may read, reflect, and be wise.

